# Incomplete information about the partner affects the development of collaborative strategies in joint action

**DOI:** 10.1371/journal.pcbi.1006385

**Published:** 2019-12-12

**Authors:** Vinil T. Chackochan, Vittorio Sanguineti

**Affiliations:** Department of Informatics, Bioengineering, Robotics and Systems Engineering, University of Genoa, Genova, Italy; Santa Fe Institute, UNITED STATES

## Abstract

Physical interaction with a partner plays an essential role in our life experience and is the basis of many daily activities. When two physically coupled humans have different and partly conflicting goals, they face the challenge of negotiating some type of collaboration. This requires that both participants understand their partner’s state and current actions. But, how would the collaboration be affected if information about their partner were unreliable or incomplete? We designed an experiment in which two players (a dyad) are mechanically connected through a virtual spring, but cannot see each other. They were instructed to perform reaching movements with the same start and end position, but through different via-points. In different groups of dyads we varied the amount of information provided to each player about his/her partner: haptic only (the interaction force perceived through the virtual spring), visuo-haptic (the interaction force is also displayed on the screen), and partner visible (in addition to interaction force, partner position is continuously displayed on the screen). We found that incomplete information about the partner affects not only the speed at which collaboration is achieved (less information, slower learning), but also the actual collaboration strategy. In particular, incomplete or unreliable information leads to an interaction strategy characterized by alternating leader-follower roles. Conversely, more reliable information leads to more synchronous behaviors, in which no specific roles can be identified. Simulations based on a combination of game theory and Bayesian estimation suggested that synchronous behaviors correspond to optimal interaction (Nash equilibrium). Roles emerge as sub-optimal forms of interaction, which minimize the need to account for the partner. These findings suggest that collaborative strategies in joint action are shaped by the trade-off between the task requirements and the uncertainty of the information available about the partner.

## Author summary

Many activities in daily life involve physical interaction with a partner or opponent. In many situations, they have conflicting goals and need to negotiate some form of collaboration. Although very common, these situations have rarely been studied empirically. In this study, we specifically address what is a ‘optimal’ collaboration and how it can be achieved. We also address how developing a collaboration is affected by uncertainty about partner actions. Through a combination of empirical studies and computer simulations based on game theory, we show that subject pairs (dyads) are capable of developing stable collaborations, but the learned collaboration strategy depends on the reliability of the information about the partner. High-information dyads converge to optimal strategies in a game-theoretic sense. Low-information dyads converge to strategies that minimize the need to know about the partner. These findings are consistent with a game-theoretic learning model which relies on estimates of partner actions, but not partner goals. This similarity sheds some light on the minimal computational machinery which is necessary to an intelligent agent in order to develop stable physical collaborations with a human partner.

## Introduction

Many activities in daily life involve coordinating our movements with those of a partner or opponent. A couple of dancers, a couple of fighters, a team of players, two workers carrying a load or a therapist interacting with a patient are just the first examples which come to mind. In all these situations, each participant in the interaction needs to know what his/her partner is doing and/or intends to do. On this basis, he/she must then select their own action [1, 2]. To do this, the two players (a ‘dyad’) may communicate verbally or non-verbally, or may watch each other. If the dyad participants are in physical contact, the forces they exchange are a rich source of information on their partner’s ongoing actions [3]. However, mechanical coupling places restrictions on individual movements, so that the choice of what to do or not do must account for what the partner is doing. Less coupling is more robust to individual inaccuracies in control—the most skilled player may still reach the goal even when the least skilled fails—thus ultimately favoring coordination, whereas more coupling may facilitate coordination only if the players have comparable skills. Further, stronger coupling provides more reliable information about partner’s actions and is therefore more informative [4].

These situations have been often studied in contexts in which there is one common and shared goal—for instance, control of isometric force [5, 6], reaching the same fixed [7, 8] or moving target [4, 9, 10], or operating a tool [3]. In these situations, dyads generally perform better [7, 9] than a person performing the same task alone. The improved performance resulting from training as a dyad also transfers to subsequent individual performance [9]. The advantage of dyad with respect to solo performance may be due to sharing of efforts—because of the signal-dependent characteristics of motor noise, less effort leads to less motor errors [11]—and/or the greater accuracy of a shared estimation of external events [9].

Specialized behaviors (i.e., ‘roles’) have been consistently observed within a dyad [12]. A common distinction is between leader and follower roles—leaders are commonly described as initiating the action and contributing most effort [6]; they also set the pace and adjust less or not at all to the partner [13]—which are commonly observed in forms of interaction in which the coupling is acoustic or visual, like joint tapping [14] and mirror games [15] but have been also reported when the coupling is physical [16].

What happens if the information about our partner is incomplete or partial? For instance, when two players can only partly see or hear each other, or when they wear padded gloves which limit tactile perception? In this case we can expect a dramatic degradation in their ability to predict their partner’s actions. However, how does such uncertainty affect the decision of which action to perform? We can expect little differences when there is a shared goal—the action to perform is the same anyway. However, if the two partners have different and partly conflicting goals, and therefore they must negotiate a collaboration, how would uncertainty affect the emerging joint coordination?

Although they are especially critical in social situations [2], these forms of interaction have received much less attention. These situations are characterized by a continuous interplay of two processes: understanding the ‘world’—dyad and environmental dynamics, partner actions and possibly partner goals—and negotiating a mutually satisfactory coordination strategy. A series of studies [17, 18] focused on ‘motor’ versions of classic non-cooperative games (like the prisoner’s dilemma)—in which position-dependent force fields encoded the player-specific costs or rewards of the interaction—or very simple motor games (e.g. tug-of-war). Bimanual versions of these tasks—in which there is only one controller—ended up in a cooperative solution. The dyad versions—two independent controllers—converged to the optimal non-cooperative solution—Nash equilibrium—a situation in which no partner can improve his/her strategy by acting unilaterally [19]. A combination of Bayesian estimation and game theory is the natural framework to address these scenarios in presence of partial information, but has never been applied to joint action involving continuous coordination with physical coupling and conflicting goals.

Collaborative behavior, if any, is the end result of learning and adaptation through repeated performance, during which the players gradually gain knowledge about dyad dynamics, the task requirements, and the partner’s actions. Nash equilibria describe optimal collaborative behaviors, but do not explain how they are achieved. Several mechanisms have been proposed [20] to account for learning a collaboration, which differ in terms of how information about the partner’s intentions and/or ongoing actions is represented. For instance, at each trial each player may form beliefs about the partner’s play and behaves rationally with regard to these beliefs—fictitious play [21, 22]. In this case, players don’t need to know about their partner’s goal (i.e. their intentions); they just need to form beliefs about how their partners will play (i.e. their actions). Alternatively, each player forms a model of the partner’s goals or, equivalently, their control law. The mechanisms through which collaborations are developed are as yet unclear and relatively unexplored.

In principle, learning to collaborate requires that both players know everything about their own and possibly their partner’s goals. In individual participants, adaptation to a novel dynamic environment, for instance a new tool, requires reliable information on the consequences of own actions; incomplete information may slow down learning and/or may affect its outcome [23–26]. In dyads, if the information about the partner is partial or incomplete, optimal collaboration may be difficult to achieve [27]. It is unclear how partial information affects establishing a collaboration in two physically interacting humans.

Here we address how ‘optimal’ collaboration can be defined when two partners have partly conflicting goals. Further, we investigate how such collaboration can be ‘learned’. Finally, we address how the learned collaboration is affected by amount and quality of information about the partner.

## Results

**Fig 1:**
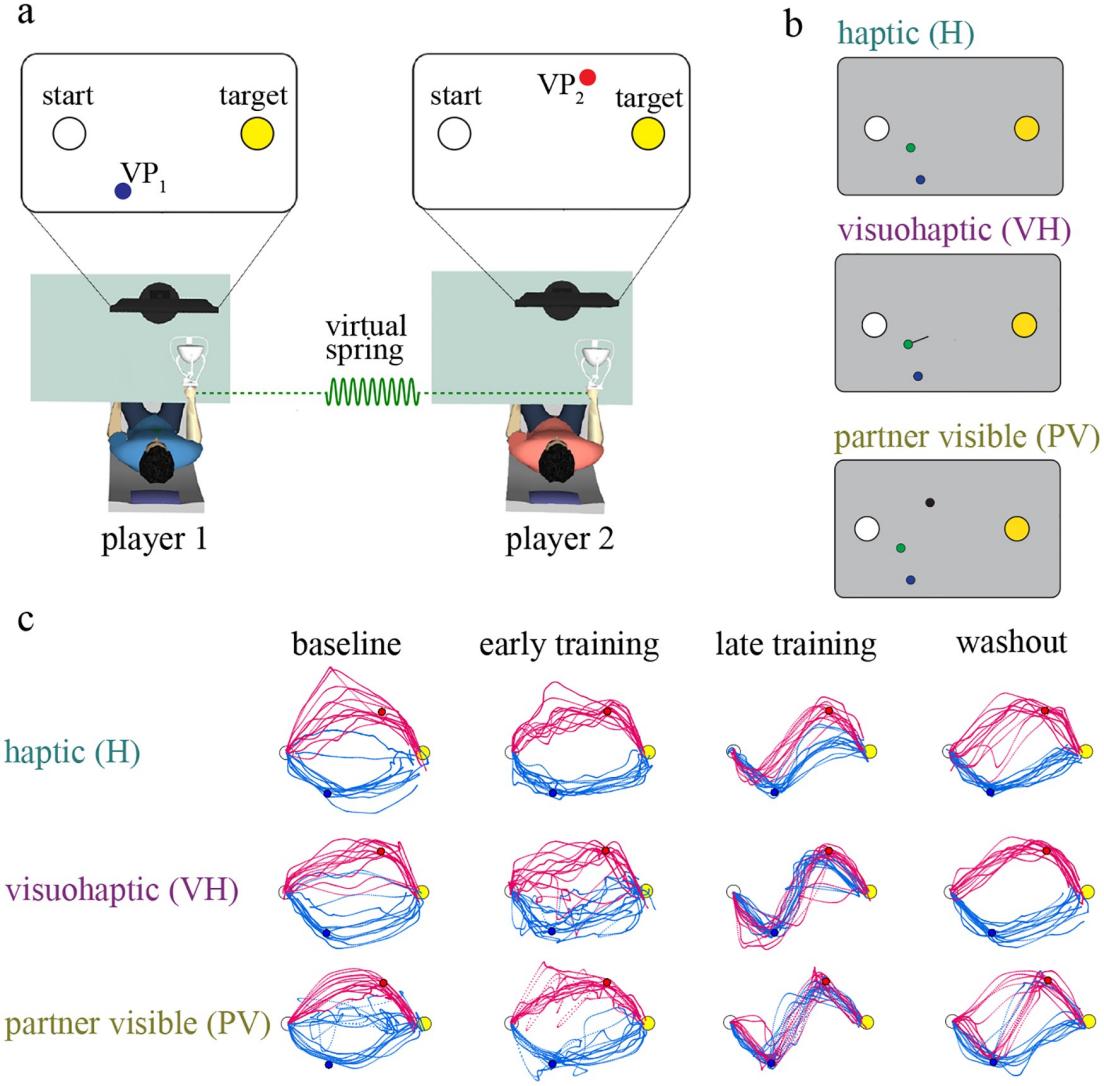
Experimental apparatus and protocol. **(a)** Players in a dyad were connected through a virtual spring. Both players were instructed to perform reaching movements in the vertical plane, between the same start point and the same target point but through different via-points (VP). Each player could only see his/her own VP, but not their partner’s. Both were instructed to keep the interaction force as low as possible during movement. The experimental protocol consisted of three phases: baseline, training and after-effect. During the baseline phase the interaction forces were turned off, and each player performed on their own (‘solo’ performance). The players were mechanically connected during the training phase, and the connection was permanently removed during the after-effect phase. **(b)** We manipulated the information available on partner’s actions by providing it either haptically, through the interaction force (Haptic group, H) or by additionally displaying the interaction force vector on the screen (Visuo-Haptic group, VH) or displaying partner’s cursor itself (Partner Visible group, PV). The yellow and white circles denote, respectively, the start and target position. The green circle is the cursor location. In the VH group, direction and magnitude of the interaction force is depicted by a line originating from the cursor. In the PV group, partner’s cursor is shown by black circle. **(c)** Movement paths in baseline (unconnected), early-training, late-training and washout phases of the experiment from three typical H, VH and PV group dyads.

We designed a novel interactive task in which—somewhat resembling the classic ‘battle of sexes’ game [28]—participants must reconcile different goals with an overall preference to stay close together. Two participants were mechanically connected but could not see each other. They were instructed to perform reaching movements with the same start and end positions, but through different via-points (VP); see Fig 1a and the Methods section for details. Both participants were also instructed to keep the interaction force as low as possible during movement. They had the option of establishing a collaboration—negotiating a path through both VPs, which would lead to a minimization of the interaction forces—or to ignore each other, by only focusing on their own goal. We manipulated the information available on partner’s actions by providing it either haptically, through the interaction force (haptic group, H); by additionally displaying the interaction force vector on the screen (visuo-haptic group, VH); or by continuously showing the partner movements (partner visible, PV); see Fig 1b.

### Collaboration in dyads and the role of information

As expected, the recorded movements were not perfectly planar. In 25/30 participants the average Z displacement over trials was less than 2 cm, with a between-participants average of 1.6 cm. Nevertheless, as the Z displacement is task-irrelevant, we excluded no data from further analysis.

In all three haptic (H), visuo-haptic (VH) and partner-visible (PV) groups, all dyads converged to stable and consistent behaviors; see Fig 1c–1e. When the connection was removed, both players quickly returned to the baseline situation. These observations are confirmed when looking at the score, the interaction force and the minimum distance from the partner’s VP. All these quantities are expected to improve if the participants establish a collaboration.

As we examine different indicators at the same time, there is indeed an increased chance of falsely rejecting the null hypothesis for some of the indicators (Type I error). As there are four indicators (score, IF, *MD*_21_, *MD*_12_) we took a Bonferroni adjusted statistical level (P = 0.05/4 = 0.0125).

**Fig 2:**
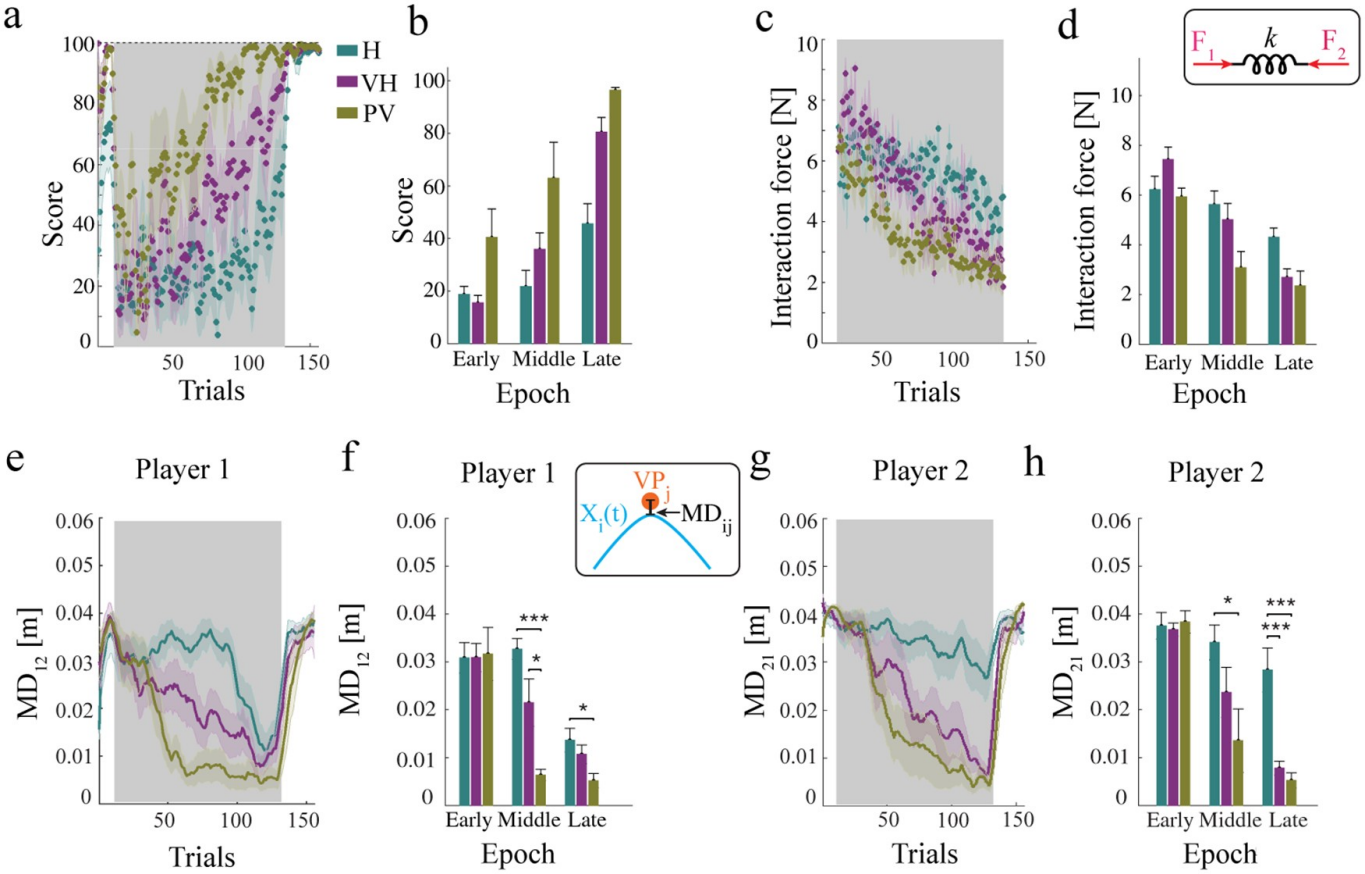
Dyads develop a collaboration, and learning depends on the amount of information available about the partner. **(a)** Temporal evolution of score over trials, for the haptic (H), visuo-haptic (VH) and partner-visible (PV) groups respectively. **(b)** Score at the beginning, middle and at the end of training. **(c)** Magnitude of the average interaction force over trials. **(d)** Interaction force at the beginning, middle and at the end of training. **(e)**, Magnitude of the distance from partner’s VP for Player 1 over trials for the H, VH and PV groups. **(f)** Minimum distances of Player 1 from his/her partner’s VP at the beginning, middle and at the end of training. **(g,h)**, As in **(e,f)** for Player 2. The areas in grey denote the training phase. All plots indicate the population means. Error bars and shaded areas denote the standard error (SE). Asterisks indicate statistically significant differences (*P < 0.05, **P < 0.01, ***P < 0.001).

The temporal evolution of score for participant pairs is summarized in Fig 2a. Overall the subject pairs improved their movement score with training—significant Time effect (*F*_2,24_ = 51; *P* < 10^−4^) and exhibited significant Group differences (*F*_2,12_ = 12; *P* = 0.0015), but we found no significant Group × Time interaction (*F*_4,24_ = 2.7; *P* = 0.0523).

The average interaction force (IF) is the main determinant of the score, and its temporal evolution exhibits a gradual decrease in all three groups—see Fig 2c. Overall, we found significant Time (*F*_2,24_ = 37.4; *P* < 10^−4^) but not Group effects (*F*_2,12_ = 6.2; *P* = 0.014), and no significant Group × Time interaction (*F*_4,24_ = 3.33; *P* = 0.026)—see Fig 2d. In summary, both score and IF improve with time, but exhibit no Group-related differences.

A similar behavior can be observed in the temporal evolution of the minimum distance from the partner’s via-point, as depicted in Fig 2e–2h. In both groups and in both players in a dyad, the minimum distance (MD) decreases over trials. The effect quickly washes out when the connection is permanently removed (after-effect phase). The magnitude of the decrease is very similar in all groups. Statistical analysis confirmed this observation. We found a significant Time effect for both Player 1 (*F*_2,24_ = 40; *P* < 10^−4^) and Player 2 (*F*_2,24_ = 36.4; *P* < 10^−4^). We also found significant Group effects (*F*_2,12_ = 7.8; *P* = 0.0067) for Player 1 and (*F*_2,12_ = 8.9; *P* = 0.004) for Player 2. We found a significant Group × Time interaction for both Player 1 (*F*_4,24_ = 5.21; *P* = 0.0036) and Player 2 (*F*_4,24_ = 4.32; *P* = 0.009).

Post-hoc analysis showed that for Player 1, in the groups (PV-H) the MD value is significantly different (lower in the PV group) in the late time (*P* = 0.0296) and also group combinations (PV-H and PV-VH) differ significantly at the middle time (*P* = 0.0002, *P* = 0.01 respectively). For Player 2, post-hoc analysis showed that group pairs (VH-H and PV-H, but not PV-VH) differ significantly at the late time (*P* = 0.0009, *P* = 0.0003 respectively) and groups (PV-H, but not VH-H and PV-VH) significantly differ at the middle time (*P* = 0.04).

In other words the three groups—specially H and PV, with VH somehow in between—differed in both magnitude and rate of decrease of their minimum via-point distances. Fig 2f and 2h summarizes the effects of learning in all three groups. This also suggests that in the H group learning is less complete at via-point 1 (Player 2) than at via-point 2 (Player 1).

Overall, the above findings suggest that in the PV group learning is faster and results in a better performance (greater score, lower interaction force, lower distance from partner VP), followed by VH and then H.

### Optimal interaction and the emergence of roles

The above results still say little on the nature of the collaboration and on how the collaboration has emerged. To address this, we developed a computational model, based on differential game theory [29], to predict the ‘optimal’ interaction behaviors—see the Methods section for details. We modeled dyad dynamics as two point masses connected by a spring. We assumed that each subject operates his/her own body—one point mass—by applying a force to it. We also assumed that each partner’s sensory system provides visual and proprioceptive information about his/her own position, plus haptic information about the interaction force, which indirectly provides information about the partner’s position.

**Fig 3:**
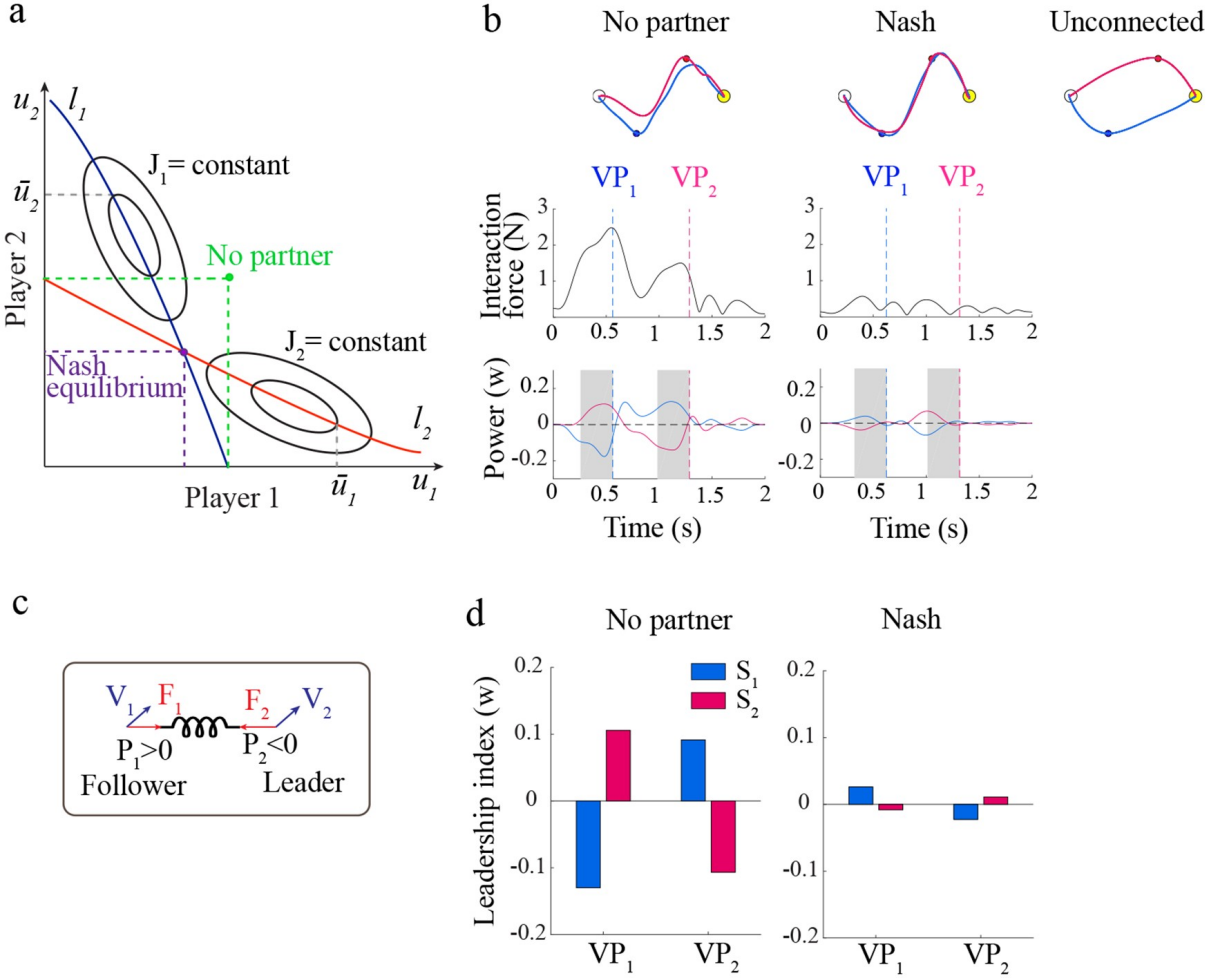
Predictions based on game theory. **(a)** Definition of Nash equilibria vs ‘no-partner’ strategies in two-players non-cooperative game. Nash equilibrium is determined by the intersection of the partners’ reaction curves—locus of optimal control action calculated for each value of the partner action [29] (blue and red lines). The ‘no-partner’ solution is determined as the optimal action calculated by each player by assuming that partner’s control is zero. **(b)** Simulated movements under Nash equilibria, ‘no-partner’ and unconnected conditions, for Player 1 (blue) and Player 2 (red). From top to bottom: movement paths, interaction force and interaction power profiles. **(c)** Interaction power, *P*_*i*_, is defined as the scalar product of the interaction force (*F*_*i*_) and the velocity(*V*_*i*_). **(d)** Leader-follower strategy in the no-partner (left) and Nash strategy (right). The plots depict the Leadership index (LI) for both players and both via-points, calculated as the average power in the 300 ms interval (in gray) just before crossing the via-point by its homologous player.

The task is specified by a pair of quadratic cost functionals (one per partner). Consistent with the score provided to participants at the end of each trial, each cost functional is a combination of distance from own via-point and interaction force with the partner, plus an effort term—see the Methods section for details. Consistent with computational models of individual movements based on optimal control [30], the interaction strategy is completely specified by a pair of controllers (one per partner). Using the model, we simulated an optimal collaboration (Nash equilibrium) [19], in which no partner can improve his/her strategy unilaterally. Another possibility is that the participants determine their control actions by assuming that they are alone in controlling the dyad dynamics. As a consequence, they focus on their own via-point and on minimizing the interaction. In this case, information on what the other partner is doing is not accounted for during action selection. This alternative scenario defines the maximum compliance with the task achievable with the minimum amount of collaboration between partners. We refer to this scenario as the ‘no-partner’ strategy—see Fig 3a.

Fig 3b summarizes the model predictions. The movement trajectories look similar in the two models, but a closer look suggests that in the no-partner case each subject actively moves toward his/her own via-point—thus behaving as a ‘leader’, but is pulled by the partner when getting closer to the other via-point—thus switching to a ‘follower’ role. This effect is clearly visible when looking at the average interaction power calculated just before crossing the via-point—see Fig 3. As a consequence, the no-partner scenario exhibits temporal delays between the via-points crossing times and a greater magnitude of interaction force and interaction power. In contrast, in the optimal (Nash) scenario the two participants approximately follow the same trajectory, by crossing each via-point at approximately the same time. Both the interaction force and the interaction power remain low over the whole movement, and there are no clear leader-follower roles. Therefore, a distinctive feature of the ‘no-partner’ scenario is the alternation of ‘leader’ and ‘follower’ roles—each participant acts as a ‘leader’ when crossing his/her own via-point, and as a ‘follower’ when crossing that of the partner. This is also reflected in the different crossing times (with respect to the ‘leader’, the ‘follower’ lags behind). In conclusion, establishing roles can be seen as a form of compensation for poor integration of the partner’s intended actions into the player’s own control strategy.

**Fig 4:**
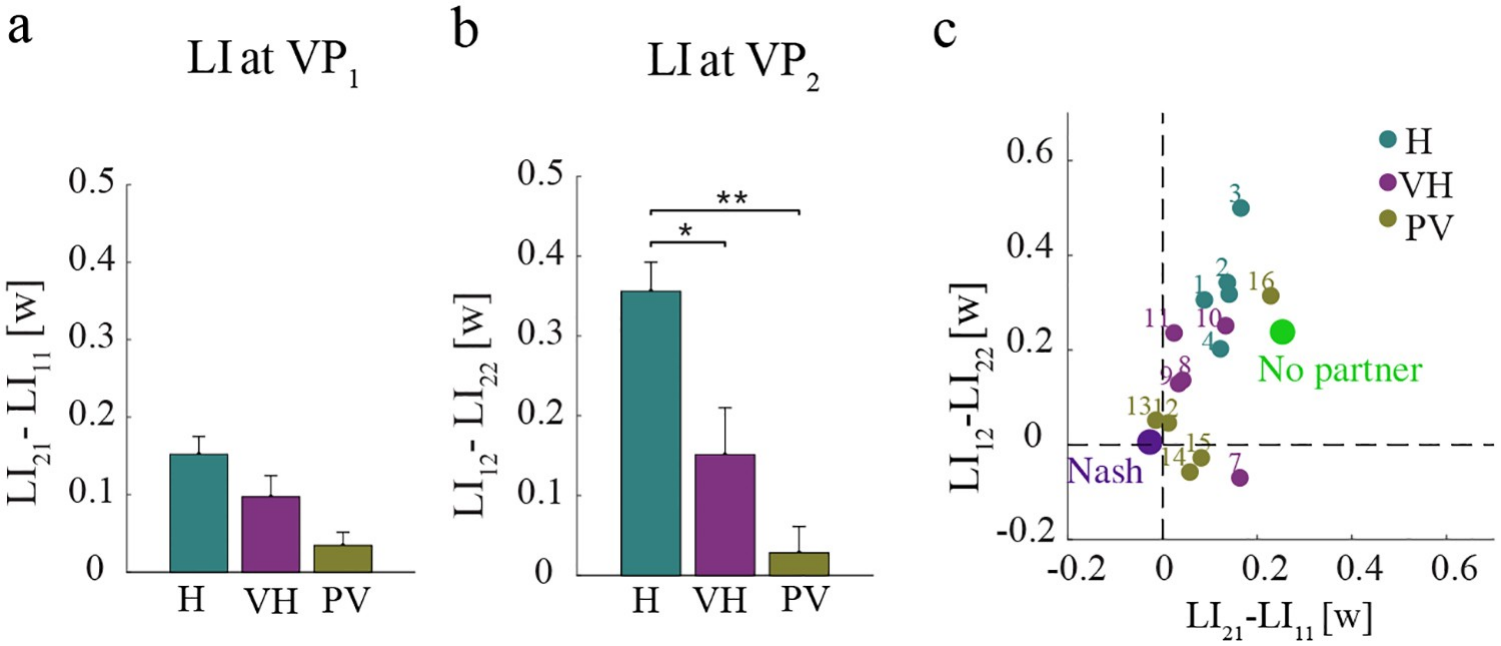
Leadership index (LI) differences at the end of training (average within the last training epoch). **(a)** Δ*LI* at *VP*_1_ is calculated as the difference between *LI*_21_ and *LI*_11_. **(b)** Δ*LI* at *VP*_2_ is calculated as the difference between *LI*_12_ and *LI*_22_. Error bars denote the standard error (SE). **(c)** Δ*LI* at *VP*_1_ and *VP*_2_, for each dyad in all three groups, compared with model predictions. The large dots denote the No partner (green) and the Nash (violet) model predictions. The plots indicate the population means. Asterisks indicate statistically significant differences (*P < 0.05, **P < 0.01, ***P < 0.001).

Based on these predictions, we looked into the emergence of distinct leader-follower roles in our experimental data at the end of the training phase. Fig 4a and 4b summarizes the leadership indices (*LI*)—average interaction power in the 300 ms interval before via-point crossing—calculated in the late epochs at *VP*_1_ (Fig 4a) and *VP*_2_ (Fig 4b).

We then calculated the difference in the interaction power for both partners at *VP*_1_ and *VP*_2_. As regards Δ*LI*_2_ = *LI*_12_ − *LI*_22_—difference in leadership indices for Player 1 and Player 2 at *VP*_2_—we found significant group differences (*F*_2,12_ = 5.84;*P* = 0.016). Planned comparison confirmed significant differences between H and VH (*t*_7.75_ = 2.63, *P* = 0.03), H and PV (*t*_7.31_ = 3.29, *P* = 0.01) but not VH and PV (*P* = 0.43). In contrast, we found no significant effects for the difference in leadership indices at *VP*_1_.

We also compared the difference in the interaction power for both partners at *VP*_1_ and *VP*_2_ in the different groups with the simulated Nash (green) and No-partner (yellow) scenarios—see Fig 4c. These results indicate that when there is limited information about the partner (group H), the players exhibit leader-follower roles near VP_2_—Player 2 behaving as a ‘leader’, Player 1 behaving as a ‘follower’. The effect decreases and tend to vanish when the amount of available information about the partner increases (from H—minimum information—to PV—maximum information). Although not statistically significant, a similar trend is observable near VP_1_—Player 1 behaves as ‘leader’, Player 2 as ‘follower’. Overall, the experimental results suggest that dyads with more available information (PV group) about the partner are closest to the optimum (Nash) scenario, whereas dyads with less reliable information (H group) resemble more the no-partner scenario.

### Learning to collaborate

**Fig 5:**
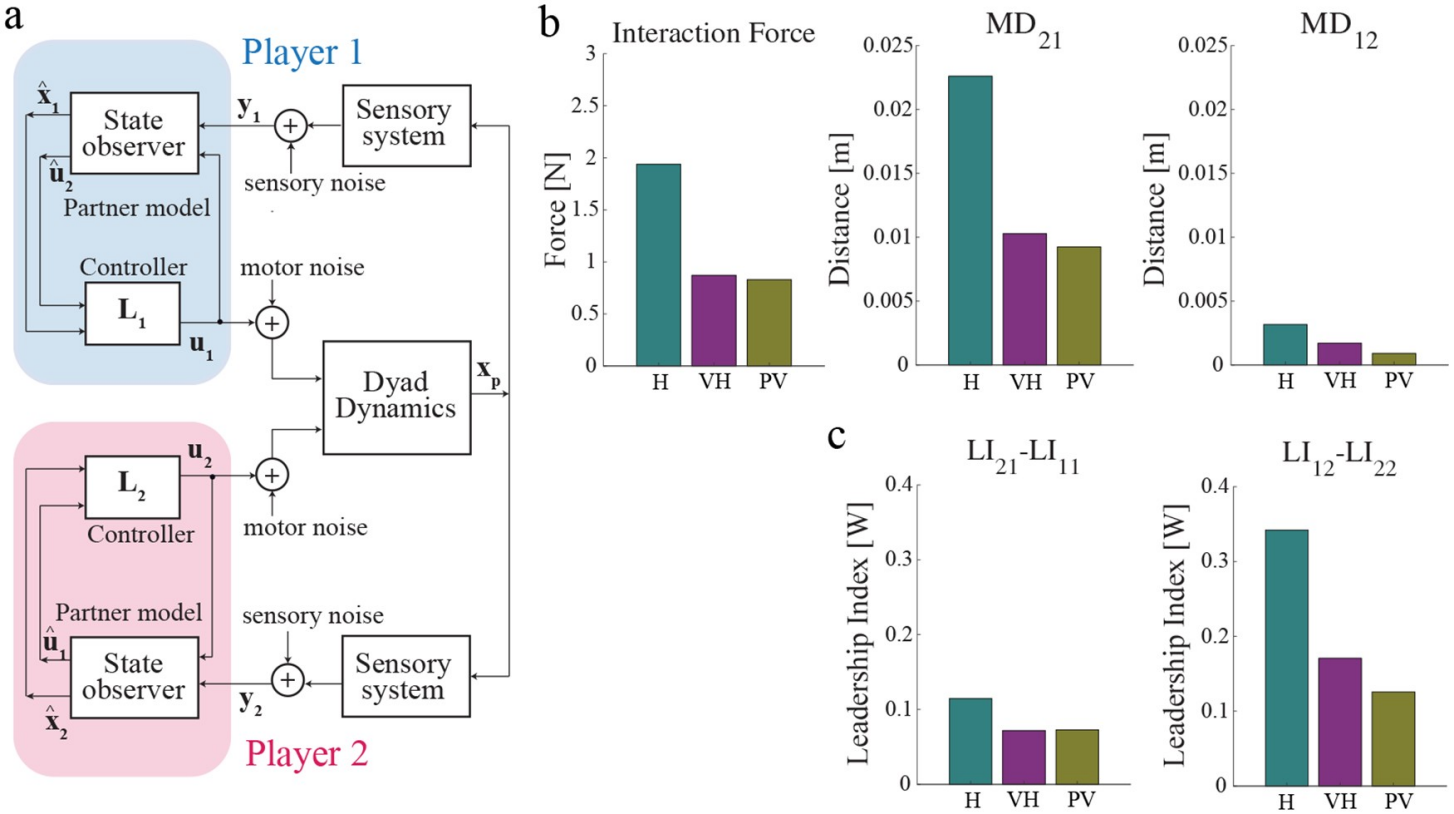
Computational model. **(a)** Two players jointly control dyad dynamics (approximated as two mass-points connected by a spring). Each player has a separate sensory system which provides information about dyad state. The controller includes a state observer, a feedback controller and an estimate of the partner’s control action (partner model). **(b)** From left to right: Interaction force and minimum distance from VP_1_ (*MD*_21_) and VP_2_ (*MD*_12_), for all three H, VH and PV scenarios. **(c)** Leadership index for player 1 and player 2 at VP_1_ (left) and VP_2_ (right) for all three H, VH and PV scenarios.

Consistent with computational models of sensorimotor control of individual movements [31], we posited that each player uses a state observer to predict the dyad state from sensory and motor information. The state observer is easily extended to also predict the partner’s control action; see Fig 5a and the Methods section.

We simulated the process of establishing a collaboration through repeated performance. The model uses a form of ‘fictitious play’ [21, 22], which requires minimal assumptions on each player’s internal representation of their partner [20]. At every trial, each player independently estimates the most likely partner’s action and incorporates it into his/her own control policy on the next trial. This model is attractive as it requires minimum information about the partner—it does not need to establish a model of the partner’s task or goals. We simulated all three scenarios (H, VH, and PV) and found—see Fig 5—that more information leads to a more Nash-like collaboration, characterized by greater synchronization and less distinct roles. This is confirmed when looking at the leadership index; see Fig 5. Switching of roles (each player leads when aiming at his/her own VP and follows when aiming at the partner VP)—which denotes a lack of consideration of partner intentions when developing their own control policy—decreases as the amount of information about the partner increases.

## Discussion

In individual sensorimotor control, uncertainty affects the estimation of the state of the body and the external environment (including tools, if any). Inaccurate state estimates may lead to inaccurate or inefficient control, may slow down learning and may affect its outcome. In joint action, uncertainty may also affect estimation of the partner’s ongoing actions (and possibly their ultimate goals). This may make establishing a collaboration more difficult or even impossible. Physical coupling is a major source of information about the partner’s behaviors. While operating a pole by pulling ropes [3], dyads produce more overlapping forces than individuals using both hands. In dyad performance, participants must keep their rope stretched to collect information about their partner’s action. If they don’t—for instance, when the rope is loose—they just have no way to coordinate their movements. These observations suggest that players in a dyad need information about their partner in order to establish a collaboration. The amount of coupling affects the quality of such information: although it may make coordination more difficult, stronger coupling provides a more reliable source of information [4].

Previous studies on joint coordination generally assumed that players had either full or complete lack of knowledge about partner’s goals, state and ongoing actions, and did not explicitly address the learning process. The present study for the first time addresses the mechanisms underlying the development of a collaboration when the information about the partner is incomplete.

### Differential game theory as general model of joint coordination

To understand how quality of information affects the learned strategies, we developed a computational model of the interaction, in which the physically coupled players form a single mechanical system, which they must jointly control. Consistent with the optimal feedback control [30] and optimal Bayesian estimation frameworks [32], which are widely used in modeling single-player sensorimotor control [31], we assumed that each participant has his/her own optimal feedback controller, completely specified by his/her own assigned task—this is called a differential game—and a state (and partner) observer, which combines sensory information and predicted dyad dynamics to estimate the dyad’s internal state and the partner actions—see Fig 3a. The model summarizes the available knowledge on the neural basis of joint coordination [31], but has been applied to the study of joint action in very limited situations, involving either discrete decisions [18]. or shared (e.g. bimanual) control [33].

Although the present study focuses on a purely motor task, differential game theory and Bayesian estimation represent a general modeling framework which can be easily extended to a broad range of tasks and situations. Game theoretic concepts are implied—although not explicitly stated—in the notions of effort sharing [34] and co-confident motion [15], but their application is relatively novel in the study of joint sensorimotor interaction. Few studies have focused on motor versions of classical games, like the prisoner’s dilemma and rope pulling [17, 18]. Although these tasks involved movements, their primary focus was on discrete decisions. These games have distinct cooperative and non-cooperative solutions, in which the players either agree or individually determine their actions. The focus here is exclusively on non-cooperative situations—in fact, in our task the cooperative and non-cooperative strategies are not distinguishable. The present study specifically uses game theory to study optimal forms of interaction and to contrast them with possible alternatives.

### All dyads gradually develop stable strategies

We argued that in physically connected dyads the development of an effective interaction is profoundly affected by information uncertainty. We focused on three groups of dyads, characterized by different amounts of information about their partner. The Haptic (H) group only relied on haptic information—from a relatively weak coupling, hence a rather unreliable information. The other groups (Visuo-Haptic, VH and Partner-Visible, PV) were provided with increasingly rich and reliable information about their partner.

We observed that dyads in all three groups gradually converge to stable interaction strategies, characterized by low interaction forces and low distances from the partner’s via-points. When two players are rigidly coupled, the development of stable coordination is relatively fast [12] or even instantaneous [8]. The rapid emergence of stable coordination in rigid coupling may be due to the fact that the partner actions are easier to predict and a joint coordination strategy is simpler to develop, at least if two players share the same goal. When the coupling is softer, learning is more gradual [4, 9]. However, dyads need more time to develop an interaction strategy when the task is more challenging [4, 10], because the coupling is softer [4, 9] or—as in the present study—when the players have different and partly conflicting goals.

Learning a collaboration is the end result of the interplay of two distinct processes: adaptation of an internal representations of plant dynamics (the state and partner observer) to changes in the environment—in our case, the onset of mechanical coupling—and the modification of the control policy to updated predictions of the partner’s actions.

### The learned interaction is influenced by the amount of available information

Nevertheless, dyads in different groups exhibited subtle but clear differences in their learned strategies. The computational model allowed to identify one specific signature of the extent to which participants use information about their partner’s actions when planning their movements. In simulations in which each player computes his/her control action by ignoring the partner, players alternate ‘leader’ and ‘follower’ roles within the same movement. Player 1 leads in via-point 1 and then follows his/her partner in via-point 2. Conversely, Player 2 follows his/her partner in via point 1 and leads at via point 2. This behavior can be interpreted in terms of the ‘minimum intervention principle’ of optimal feedback control [30]. For Player 1, via-point 2 is task-irrelevant and therefore getting close to this point is not controlled explicitly; vice versa for Player 2.

These predictions are confirmed by our experimental results. We found that dyads characterized by less reliable partner information (group H) are qualitatively very similar to the predicted ‘no-partner’ behaviors. In contrast, leader-follower patterns tend to disappear when information is more reliable. The dyads with more information about the partner (PV group) exhibit a form of collaboration which is very close to optimal (Nash strategy). The dyads in the VH group lay somehow in between. Consistent with these observations, leader-follower roles are observed in imitation tasks, like joint tapping [14] or mirror games [15], when partners lack feedback about their partner’s actions.

Are the roles affected by training? In a study involving joint generation of isometric forces [6], leader-follower relations were observed in novice-experienced pairs but were not affected by practice. In contrast, in both simulations and experiments, we found that roles evolve with the knowledge gained about the partner. As a consequence, in all groups, early trials exhibit distinct roles. In high-information dyads (VH and PV group) roles gradually disappear and the collaboration strategy is closer to Nash equilibrium. In contrast, in low-information dyads (H group), roles are preserved until the end of training. Overall, these observations suggest that leader-follower strategy is a sub-optimal form of collaboration, consequent to an incomplete co-activity. In conclusion, incomplete information prevents more efficient forms of collaboration, which require reliable estimates of the partner’s state and current actions. The notion that roles emerge as a sub-optimal form of collaboration when partner information is uncertain or absent is consistent with the recent observation [13] that roles assigned a priori tend to be detrimental to coordination, unless they are determined by the lack of perceptual access to partner’s actions.

### How is collaboration learned?

We used the computational model not only to predict optimal behaviors, but also to understand how these behaviors are learned. We suggest that the development of optimal collaboration may be accounted by fictitious play [21], an iterative process in which two players play the game repeatedly. In every round each player determines its best response against the empirical strategy distribution of his/her partner.

In our implementation of fictitious play, at each iteration each player determines his/her optimal controller by accounting for the partner’s most likely action, assumed to correspond to the action estimated on the previous trial. The optimal controller has a feedback part, driven by the estimated plant state, and a feedforward part which reflects the partner’s predicted action.

Like plant state, prediction of the partner’s action mostly relies on the available sensory information. Our learning model specifically predicts that dyads converge to a Nash equilibrium if players have reliable information about their partner. When information uncertainty increases, action prediction becomes less reliable. In this case, consistent with the experimental findings, the dyad establishes a pragmatic form of collaboration, which largely ignores what the partner does/is doing.

The model also captures another empirical finding, i.e. an asymmetry of behaviors between Player 1—aiming at VP_1_—and Player 2—aiming at VP_2_ in the low-information group (H). In particular, Player 2 is systematically less accurate at reaching VP_1_, than Player 1 at reaching VP_2_—in other words, MD_21_ tends to be greater than MD_12_; see Fig 2e–2h. Conversely, the leadership index tends to be greater in VP_2_ than in VP_1_; see Fig 4a and 4b. The model captures both asymmetries, between Players and/or VPs—see Fig 5b and 5c. In particular, the simulations suggest that the early part of the trajectory, including the portion around VP_1_, exhibits more variability because the players initially have poorer estimates of their partner’s motor command. In empirical results this effect is further emphasized, possibly because the players don’t start their movements exactly at the same time (this is not accounted for in the model).

This model adds computational substance to previous models of joint action, which only address the representation level [35] or the dynamics of the coupling [36]. Our proposed model also has possible technological implications, as it sheds some light on the minimal computational machinery which is necessary to an intelligent agent in order to develop stable physical collaborations.

### Do players understand each other’s intentions?

In our proposed control model we show that reliable estimation of partner’s action can be achieved through a simple extension of the state observer. In other words, we assume that partner’s action recognition is obtained through a optimal (in Bayes’ sense) combination of predictions and observations. Our results provide no direct information on the possible neural substrates of action observation, which is often associated with the mirror neuron system [37]. However, our prediction that estimation of partner actions during joint action is no different from estimating other aspects of plant state points at a role of the cerebellum—which is involved in the representation of body and environment dynamics—in conjunction with brain areas like the superior temporal sulcus that have been associated to action observation [2].

The empirical findings and the model simulations are consistent with the notion that each player establishes a model of the partner’s current actions. Similarly, in a sensorimotor coordination game, human players against computer partners adapted their behavior to the partner’s willingness to cooperate [38]. One crucial question in joint action is whether and to what extent the two partners within the dyad develop a deeper form of understanding, related to their partner’s goal. While there is some evidence [39] that participants in a dyad do develop and maintain models of their partner goals even when not strictly necessary (e.g. when acting individually), and even when this is detrimental to individual performance, other studies [40, 41] report little evidence of players modeling their partners’ goals or intentions. Our conclusions are also consistent with Noy et al. [42], claiming that coupled forward models are necessary for producing ‘co-confident motion’ without a leader and a follower. This conclusion seems to imply that partners need to predict each other’s actions.

In our study, the gradual decrease in the leadership indices suggests that participants incorporate information about their partner into their motor plans. Our experiment was carefully designed so that participants had no explicit clue on the partner’s task. After the end of each experiment, the participants gave no consistent answer when asked what they thought the partner was doing. This is consistent with the fictitious play model of learning, which does not require to model the partner’s task explicitly—this is what should properly be referred as intention—but simply requires to account for the partner’s most likely action, inferred from previous trials. Therefore, both experiments and simulations suggest that at least in this task, players only need minimal information about their partner to converge to quasi-optimal behaviors (Nash equilibrium). Nevertheless, it may be entirely possible that in dyads of the PV group seeing the partner’s cursor could have provided informative cues about their task, especially if players assumed that their partner’s task was similar to theirs (i.e., moving to a target via a point in between). Therefore, our findings do not rule out the possibility that in other more complex forms of interaction the players estimate their partner goals. Indeed, the proposed Bayesian model of action estimation can be easily extended to estimation of partner’s goals. Future experiments, possibly involving generalization to other tasks or interacting with a virtual partner will be necessary to clarify this important point.

### Study and model limitations

Each experimental group involved a total of ten participants, i.e. five dyads. Dyads performance was quite consistent within each group. Nevertheless, due to the limited sample size, the generalizability of the above findings must be taken cautiously.

Optimal feedback control models of individual sensorimotor control [30] typically assume that process noise increases its magnitude with that of the motor command—signal-dependent or multiplicative noise. Multiplicative noise is ubiquitous in the motor system. To keep the model simple, as in related studies [4] we only included a large, additive Gaussian noise term. This may have underestimated the between-trial variability. In spite of this limitation, the model predictions closely resemble empirical observations. However, the model can be extended where necessary by using results in differential game theory in which multiplicative noise is used in differential games with both finite [43, 44] and infinite horizon [45].

## Materials and methods

### Experimental apparatus and task

Each experiment involved one pair of participants (a dyad). Participants sat in front of two separate computer screens and grasped the handle of a three-dimensional haptic interface (Novint Falcon). They could not see or hear each other, and were not allowed to talk. The experimental apparatus is depicted in Fig 1. The participants were instructed to perform reaching movements in the vertical plane, between the same start point (displayed as a white circle, ⊘ 1 cm) and the same target point (yellow circle, ⊘ 1 cm), but through different via-points. In a reference frame centered on the robot workspace (one for each participant), with the X axis aligned with the left-right direction and the Y axis aligned with the vertical direction, the start point was placed in the (-5, 0, 0) cm position and the target point was placed in the (5, 0, 0) cm position. Hence the start and the target point had a horizontal distance of 10 cm. The current positions of the end effector were continuously displayed to each participant as a ⊘ 0.5 cm circular cursor on his/her respective screens. The participants were also instructed to keep their movements as planar as possible, i.e. by keeping the Z component of end effector position within a ±4 cm range with respect to the XY (vertical) plane. To encourage them to do so, the cursor was displayed in green if the depth was within limits, in red otherwise.

A trial started when both participants placed their cursor inside the start region. Then the target and a via-point (⊘ 0.5 cm circle) appeared. The via-points were different for the two participants and were placed, respectively, at locations VP_1_ = (-3,-2,0) cm and VP_2_ = (3,2,0) cm. The haptic interfaces generated a force proportional to the difference of the two hand positions:

$$\begin{array}{c} F_{1}=-k\left(x_{1}-x_{2}\right) \end{array}$$ (1)

$$\begin{array}{c} F_{2}=-k\left(x_{2}-x_{1}\right) \end{array}$$ (2)

with *k* = 150 N/m. Hence, the two participants were mechanically connected.

The participants were also told that they might experience a force while performing the task, and were instructed to keep this force to a minimum. We decided to introduce this additional requirement because of a technical limitation of our robots, which are unable to generate large forces. With a low stiffness. the haptic perception of the interaction force is less reliable, and introducing an explicit requirement on low interaction forces made sure that all players were provided with exactly the same task requirements irrespective of the information they had available about their partner.

At the end of each movement, each subject received a 0-100 reward, calculated as a function of the minimum distance of their movement path from his/her own via-point and of the average interaction force:

$$\begin{array}{c} {\text{score}}_{i}=\frac{100}{1+\exp^{h(d_{i}-d_{0})}} \end{array}$$ (3)

where $d_{i}=d_{{VP}_{i}}+c\cdot d_{12}$ and *i* = 1, 2. The quantities $d_{{VP}_{i}}$ and *d*_12_ are, respectively, the minimum distance between the movement trajectory and the subject’s own ‘via-point’ (*VP*_*i*_) and the average distance between the two participants’ hand positions. In the disconnected trials we took *c* = 0, i.e. the score only depended on how close the participants got to their own via-point. Parameters *h* and *d*_0_ were calculated so that the score was maximum (100) for *d*_*i*_ ≤0.005 m (i.e., the VP radius), and minimum (0) for *d*_*i*_ ≥ 0.02 m. Audio cues were provided at the start and end of the movements. To encourage participants to establish a collaboration, in trials in which the two participants were mechanically connected we took *c* = 0.5, so that in order to get a maximum score participants also had to keep their relative distance as low as possible. The participants were encouraged to aim at maximizing this score. Specifically, they were told that performance depends on how close they would get to the via-point. They were also warned that the perceived force magnitude also affects the score. It should be noted that the score is only meant as a reinforcement signal to sustain players’ motivation and to speed up learning. As such, it specifically focuses on few aspects of the task (pass through the via-point; keep the interaction forces low). Other task requirements, like reaching the target, require no reinforcement.

The interaction force and the calculated score only took into account the X and Y components of the trajectories. Hence the Z component of the end effector can be considered as task-irrelevant. To encourage participants to maintain an approximately constant movement duration, after each movement a text message on the screen and changes in the color of the target (either green or red) warned the participants if the movement was either too fast (duration < 1.85 s) or too slow (duration > 2.15 s). However, the participants received no penalization if their movement duration did not remain within the recommended range.

The participant pairs were randomly assigned to three groups depending on the feedback provided about the interaction force. In the haptic (H) group, interaction could only be sensed haptically. In the visuo-haptic group (VH), interaction force (magnitude, direction) was also displayed as an arrow attached to the cursor (scale factor: 10 N/cm). In the partner-visible group (PV), the participants could see their partner’s cursor. Therefore, these participants have a more reliable information about partner’s movement. The experiment was organized into epochs of 12 movements each. The experimental protocol consisted of three phases: (i) baseline (one epoch), (ii) training (ten epochs) and (iii) after-effect (two epochs) for a total of 13 × 12 = 156 movements. During the baseline phase the interaction forces were turned off, and each participant performed on their own. During the training phase the participants were mechanically connected. During this phase, in randomly selected trials (catch trials) within each epoch (1/6 of the total, i.e. 2 trials per epoch) the connection was removed. The connection was permanently removed during the after-effect phase. During the training phase the participants had the option to establish a collaboration—negotiating a path through both via-points, which would lead to a minimization of the interaction forces and a maximum score for both—or to ignore each other—each partner would only focus on their own via-point and on maximizing his/her own score. We developed a custom software application using CHAI3D, an open source software environment for control of haptic devices [46].

### Subjects

A total of 30 subjects participated in this study, recruited among the graduate and undergraduate students of University of Genoa. All participants were right-handed, as assessed using the Edinburgh Handedness Inventory [47], naïve to the task and with no known neurological or motor impairment at the upper limb. From the list of participants, we formed 15 dyads with similar body size (assessed through the body mass index) which were randomly assigned to the H (25 ± 5 y; 9 M + 1 F), VH (24 ± 3 y; 8 M + 2 F) and PV groups (24 ± 3 y; 6 M + 4 F). The two participants within the same dyad were randomly labelled as, respectively, Player 1 and Player 2.

The research conforms to the ethical standards laid down in the 1964 Declaration of Helsinki that protects research participants and was approved by the competent ethical committee (Comitato Etico Regione Liguria). Each subject signed a consent form conforming to these guidelines.

### Data analysis

Hand trajectories and robot-generated forces were sampled at 100 Hz and stored for subsequent analysis. The data samples were smoothed by means of a 4th order Savitzky-Golay filter with a 370 ms time window. We used the same filter to estimate velocity and acceleration. We identified the start and end times of each trajectory as the time instants at which the speed crossed a threshold of 2 cm/s.

In the analysis, we specifically focused on the temporal evolution of the trajectories and on signs of collaboration between players within the same dyad. Collaboration can be characterized in terms of both movement kinematics and movement kinetics. The average interaction force (IF) is calculated as $IF=\frac{1}{N}\sum_{t}∥F\left(t\right)∥$, where *F*(*t*) is equal and opposite for the two partners in the dyad—see Eq 2. Less interaction force would point at a greater collaboration.

A sign of collaboration is that each player, while passing through his/her own via-point, also gets very close to his/her partner’s. This can be quantified in terms of the Minimum via-point Distance (MD_*ij*_), defined as the minimum value of the distance of Player *i* to the *j*-th via-point: $MD_{ij}={\text{min}}_{t}\left\|x_{i}\left(t\right)-x_{VP_{j}}\right\|$ with *i*, *j* = 1, 2. If *i* ≠ *j* this quantity reflects how close each player gets from his/her partner’s via-point.

Looking at the power developed by each player would provide information on whether the players move actively, or are passively pulled by their partner through the mechanical coupling. To quantify this, we calculated the power (*P*_*i*_), defined as the scalar product of the interaction force *F*_*i*_(*t*) and the velocity vector *v*_*i*_(*t*) of each of the players. At a given time, a negative power would mean that the player is moving against the mechanical coupling. We operationally define this behavior as that of a ‘leader’. Conversely, a positive power would indicate that the player is being pulled toward the other, i.e., he/she is behaving as a ‘follower’—see Fig 3c. We specifically focused on the average power calculated in the 300-ms interval taken just before the crossing of each via-point. We denote as LI_*ij*_ this value for the *i*-th player and the *j*-th via-point. For each dyad we also calculated a cumulative leadership index associated to either VP_1_ and VP_2_, respectively calculated as ΔLI_1_ = LI_21_ − LI_11_ and ΔLI_2_ = LI_12_ − LI_22_. Large positive values of these quantities denote greater role specialization around that via-point.

All quantities (trajectories, velocities, interaction force, interaction power) were computed from the recorded movements by only taking into account the X and Y components of hand trajectories and interaction forces. Therefore, the results do not rely on planarity of the movements, which was not explicitly reinforced.

We expect that task performance at players and dyad level evolves with time (learning) and is affected by the amount of information each player has available about his/her own partner. To test this, for all the above indicators we ran a repeated-measures ANOVA with group (H, VH, PV) and Time (early—training epoch 1, middle—epoch 6 and late—epoch 11) as factors. If a significant main effect was found, Tukey’s honest significant difference (HSD) post-hoc test was used to further examine the differences. All data were analyzed using MATLAB (R2017b) and the statistical tests were performed using R (R Studio 1.1). Statistical significance was considered at *P* < 0.05 level for all tests. Wherever necessary, we used Bonferroni correction to account for multiple comparisons.

### Computational model

We compared the observed movements with simulations from a computational model, whose purpose was to predict the ‘optimal’ behaviors and how they depend on the information available about the partner. We approximated the dyad dynamics and the participants’ sensory systems as a linear discrete-time dynamical system with additive Gaussian noise in both motor commands and sensory measurements.

#### Dyad dynamics

We assume there is one single plant, reflecting dyad dynamics—i.e. both players’ body dynamics and their mechanical interaction. Dyad dynamics was approximated as a pair of point masses connected by a spring. We assumed that each participant operates his/her own point mass by applying a force to it. The dyad state trajectory, *x*(*t*), is determined by both partner’s control commands, *u*_1_(*t*) and *u*_2_(*t*). Therefore, dyad dynamics can be described by a discrete-time linear dynamical system with two inputs:

$$\begin{array}{c} x\left(t+1\right)=A\cdot x\left(t\right)+B_{1}\cdot [u_{1}\left(t\right)+\eta_{1}\left(t\right)]+B_{2}\cdot [u_{2}\left(t\right)+\eta_{2}\left(t\right)] \end{array}$$ (4)

The state variable *x*(*t*) accounts for position, velocity and muscle activation dynamics of both partners (eight variables per partner). For notational convenience, we also added the desired final position (two dimensions) and the two via-points (two 2-dimensional vectors) to the state vector; see the S1 File. for details. We also assumed that both inputs (one two-dimensional vector per player) are affected by motor noise, assumed to be Gaussian and zero-mean: $\eta_{i}\left(t\right)∼N\left(0,\Sigma^{\eta_{i}}\right)$, with *i* = 1, 2. The noise covariance matrices $\Sigma^{\eta_{i}}$, *i* = 1, 2 reflect the non-deterministic component of plant dynamics.

#### Sensory system

Each partner’s sensory system provides visual or proprioceptive information about his/her own position and via-point, the target and haptic information about the interaction force:

$$\begin{array}{c} \begin{array}{c} y_{1}\left(t\right)=H_{1}\cdot x\left(t\right)+v_{1}\left(t\right)\\ y_{2}\left(t\right)=H_{2}\cdot x\left(t\right)+v_{2}\left(t\right) \end{array} \end{array}$$ (5)

where *v*_1_(*t*) and *v*_2_(*t*) are zero-mean, Gaussian sensory noise processes: $v_{i}\left(t\right)∼N\left(0,\Sigma_{i}^{v}\right)$, with *i* = 1, 2. Eq 5 implies that each player’s sensory system has a (partial and noisy) knowledge of the whole plant state, determined by model parameters *H*_*i*_ and $\Sigma_{i}^{v}$, *i* = 1, 2.

#### Task specification

Consistent with the optimal control model of sensorimotor control [30], the players’ goals are specified by a pair of cost functionals (one per player):

$$\begin{array}{c} \begin{array}{c} J_{1}[u_{1},u_{2}]=\sum_{t=1}^{T-1}[x{\left(t\right)}^{T}\cdot Q_{1}\left(t\right)\cdot x\left(t\right)+u_{1}{\left(t\right)}^{T}\cdot R_{1}\left(t\right)\cdot u_{1}\left(t\right)]+x{\left(T\right)}^{T}\cdot Q_{1}\left(T\right)\cdot x\left(T\right)\\ J_{2}[u_{1},u_{2}]=\sum_{1=1}^{T-1}[x{\left(t\right)}^{T}\cdot Q_{2}\left(t\right)\cdot x\left(t\right)+u_{2}{\left(t\right)}^{T}\cdot R_{2}\left(t\right)\cdot u_{2}\left(t\right)]+x{\left(T\right)}^{T}\cdot Q_{2}\left(T\right)\cdot x\left(T\right) \end{array} \end{array}$$ (6)

Each cost functional has an error and an effort term, respectively depending on the dyad state and on the player’s control action. Due to the mechanical coupling within the dyad, the movements of each player are affected by the control action of his/her partner. Therefore, each cost functional depends on the control action of both players. This is called a linear-quadratic discrete-time differential game [29]. In simulations, we set the cost functionals to reflect the task requirements for each player. This includes getting to the target and staying there, passing through their respective via-points and minimizing the interaction force; see S1 File for a detailed description of the implementation of the cost functionals.

#### Optimal controllers

We first used the model to reproduce two ideal situations. The interaction strategy can be derived from the above pair of feedback controllers. Assuming that the two players have a perfect knowledge of the plant state, the dyad’s control system can be modelled as a pair of feedback controllers, one per each player—see below and S1 File for details.

$$\begin{array}{c} u_{i}\left(t\right)=-L_{i}\left(t\right)\cdot x\left(t\right) \end{array}$$ (7)

with *i* = 1, 2. We also assume that each player autonomously determines his/her own control policy, {*L*_*i*_(⋅)}, with no explicit agreement with his/her own partner—which in game theory is called a non-cooperative scenario.

The controller gains in the optimal controller can be calculated on the basis of the assumptions that each player makes on his/her partner. We specifically focused on two different scenarios: (i) each partner has a perfect knowledge of their partner’s control policy—this corresponds to the optimal non-cooperative solution; and (ii) each player completely ignores their partner when determining his/her control policy—these will be referred as the ‘no-partner’ solution.

The optimal non-cooperative solution—Nash equilibrium [19]—corresponds to a situation in which each partner cannot improve his/her strategy unilaterally; see Fig 3b. A pair of control policies $\left[u_{1}^{*},u_{2}^{*}\right]$ is a Nash equilibrium if none of the players can achieve a lower cost magnitude by unilaterally changing his/her own control policy:

$$\begin{array}{c} J_{1}[u_{1}^{*},u_{2}^{*}]\leq J_{1}[u_{1},u_{2}^{*}]\;\;\;\forall u_{1}\neq u_{1}^{*} \end{array}$$ (8)

and similarly

$$\begin{array}{c} J_{2}[u_{1}^{*},u_{2}^{*}]\leq J_{2}[u_{1}^{*},u_{2}]\;\;\;\forall u_{2}\neq u_{2}^{*} \end{array}$$ (9)

Nash equilibrium strategies of feedback type can be computed in terms of differential game theory [29] in the same way optimal feedback control theory has been used to predict optimal movements of a single human [30]. Nash equilibria represent the optimal form of collaboration which two partners can achieve when independently planning their actions (i.e. non-cooperatively). Importantly, these strategies require that each player has a perfect knowledge of his/her partner’s cost function.

The ‘no-partner’ strategy implies that the two partners do not collaborate at all, in the sense that they ignore each other when determining their control policy. We modelled this situation by assuming that each subject develops a control strategy by considering the partner’s control as noise. In this case, the problem reduces to separately developing two independent optimal Linear Quadratic Gaussian (LQG) controllers. In this case, each player only needs to know his/her own cost function.

For the task under study we calculated both controller gains—see the S1 File for details—and simulated the resulting trajectories by assuming perfect state information; see Fig 3.

#### State observer and partner model

The control strategies are not the only determinants of behavior. The above calculations of the optimal control policy assume that each player has a perfect information on the plant state vector, *x*(*t*).

However, both partners have incomplete knowledge of the system state. In a single-player situation, player *i* may predict the state at time *t* by combining prior knowledge of dyad dynamics (forward model), including a copy of his/her own motor command, *u*_*i*_ (efferent copy) with his/her own sensory information, *y*_*i*_(*t*). The combination of using own sensory feedback and forward model to estimate the current state—usually referred as sensorimotor integration—is known as state observer. A state observer relies on the optimal combination of prediction and correction. Prediction requires an accurate model of dynamics and a copy (efferent copy) of his/her own motor command. Correction is driven by the information provided by the sensory system [32].

In the case of linear systems with Gaussian noise, the Kalman algorithm is an optimal (Bayesian) solution to this state estimation problem. The posterior estimate of the next state, ${\hat{x}}^{+}\left(t+1\right)$, has the general structure:

$$\begin{array}{c} {\hat{x}}_{i}^{+}\left(t+1\right)={\hat{x}}_{i}^{-}\left(t+1\right)+K_{i}\left(t+1\right)\cdot \left[y_{i}\left(t+1\right)-H_{i}\cdot {\hat{x}}_{i}^{-}\left(t+1\right)\right] \end{array}$$ (10)

The two components of the observer are, respectively, the ‘prediction’ and the ‘correction’ (or ‘innovation’) terms.

In particular, the optimal ‘prior’ prediction of the next state by subject *i*, ${\hat{x}}_{i}^{-}\left(t+1\right)$—i.e. the estimation obtained before the sensory feedback *y*_*i*_(*t* + 1) is measured—is given by:

$$\begin{array}{c} {\hat{x}}_{i}^{-}\left(t+1\right)=A\cdot {\hat{x}}_{i}^{+}\left(t\right)+B_{i}\cdot u_{i}\left(t\right) \end{array}$$ (11)

The Kalman gain *K*_*i*_(*t*), *t* = 1, ⋯, *T* of the innovation term is determined by the Kalman iterative algorithm and reflects the trade-off of the reliability of the prediction and correction terms. If the prediction term is highly reliable—i.e., if we have a good knowledge about plant dynamics—the innovation term will add little to the state estimation and the Kalman gain will be small. In contrast, if the sensory input is highly reliable the Kalman gain will be large and the state estimation will be largely determined by the innovation term.

When there are two players acting on the same plant, unbiased estimation of the plant state also requires the partner’s input, *u*_−*i*_(*t*) which is generally unknown. Several authors—among others, [48]—have proposed general solutions to the problem of joint estimation of input and state when no prior information about the input, thus resulting in a generalisation of the Kalman algorithm. The main idea is that sensory measurements at time *t* contain information about the unknown input at time *t* − 1 and earlier. Under the reasonable assumption that partner’s input is smooth, here we formulate the problem of estimating *u*_−*i*_(*t* − 1) as a simple extension of the Kalman algorithm. The smoothness assumption of the partner’s input can be formalised in the following expression:

$$\begin{array}{c} u_{-i}\left(t+1\right)=A_{u}\cdot u_{-i}\left(t\right)+\varepsilon_{-i}\left(t\right) \end{array}$$ (12)

where 0 < *A*_*u*_ < 1 and $\varepsilon_{-i}\left(t\right)∼N\left(0,\Sigma_{-i}^{\varepsilon }\right)$. Eq 12 corresponds to the prior belief that the partner’s input is a low-pass filtered Gaussian noise.

We can now define, for each player, an augmented state: *X*_*i*_(*t*) = [*x*(*t*), *u*_−*i*_(*t* − 1)]^*T*^. Eqs 4 and 12 can be grouped together as:

$$\begin{array}{c} X_{i}\left(t+1\right)=[\begin{array}{cc} A & B_{-i}A_{u}\\ 0 & A_{u} \end{array}]\cdot X_{i}\left(t\right)+[\begin{array}{c} B_{i}\\ 0 \end{array}]\cdot u_{i}\left(t\right)+w_{i}\left(t\right) \end{array}$$ (13)

where $w_{i}\left(t\right)=N\left(0,\Sigma_{i}^{w}\right)$ and $\Sigma_{i}^{w}=\text{diag}\left(B_{i}\Sigma_{i}^{\eta }B_{i}^{T},\Sigma_{-i}^{\varepsilon }\right)$.

A key assumption of our model is that each player has a state observer for the above augmented dynamics; see Eq 10. Hence the player’s state observer combines information on plant dynamics and own sensory information to predict both dyad dynamics and partner’s input.

In particular, estimation of partner’s input combines prediction, expressed by Eq 12, with a correction term which reflects the sensory information. Parameters *A*_*u*_ and $\Sigma_{-i}^{\varepsilon }$ define the prior knowledge on partner’s input. The ‘partner’ portion of the Kalman gain regulates the importance of the prediction and correction terms. If sensory information is highly reliable, the contribution of prediction is neglectable. Conversely, if sensory information is less reliable, the estimate is mostly driven by the prediction. As a consequence, we expect that more uncertain is the sensory information, less reliable will be the partner’s action estimation.

In summary, the above formulation assumes that in joint action each player has his/her own sensory system, control policy and state observer. The latter also includes an internal representation of the partner’s input. We will refer to this as the player’s ‘partner model’. In other words, the model assumes that joint action requires that each player infers what the other partner is doing. The model—summarised in Fig 3a—only constitutes a reference to understand the consequences of the different assumptions.

#### Learning through fictitious play

In the previous sections we introduced a general optimality framework to account for dyad behaviours. In the case of perfect information about the plant, the task and the partner, Nash equilibrium is the predicted optimal behaviour if the two players act independently in determining their respective control policies (non-cooperative play). However, Nash equilibria describe the optimal collaborative behaviour but do not tell us how do players achieve it. Collaborative behaviour results from repeated task performance, during which the players gradually gain knowledge about dyad dynamics, the task requirements, and the partner’s actions. This suggests that collaboration, if any, is a result of learning and adaptation.

One possible solution of the problem of iteratively calculating a Nash equilibrium is represented by the classical learning process known as fictitious play or as the Brown-Robinson learning process, originally introduced by [21] as an algorithm for finding the value of a zero-sum game, and first studied by [22]. In fictitious play, two players play the game repeatedly. After arbitrary initial moves in the first round, in every round each player determines its best response against the empirical strategy distribution of his/her partner.

In fictitious play, strict Nash equilibria are absorbing states [20]. In other words, if at any time period all the players play a Nash equilibrium, then they will do so for all subsequent rounds. Further, if fictitious play converges to any distribution, those probabilities correspond to a Nash equilibrium of the underlying game. Convergence does not occur in general, but many authors have identified classes of games for which such convergence holds; see [49] for review.

Fictitious play has two basic properties: (i) It is only adequate if the partner uses a stationary strategy; (ii) It does not require that each player knows the partner’s task as it only requires a model of the strategy distribution. In other words, players don’t have to know anything at all about their opponent’s payoffs. All they do is to form beliefs about how their opponents will play [20]. Alternatively, players need to incorporate beliefs about opponent’s strategies or require players to have a ‘model’ of the game. While many studies agree that humans can form ‘models’ of their opponents and/or they ‘understand’ their intentions, the exact nature of these models remains elusive. Here we use fictitious play as it represents the simplest form of ‘partner model’.

Within our modelling framework we implemented a simplified version of fictitious play by assuming that each player ‘sees’ a plant that incorporates partner’s input estimated at the previous trial. For Player *i*, the augmented dyad dynamics is defined as:

$$\begin{array}{c} \left[\begin{array}{c} x(t+1)\\ 1 \end{array}]=[\begin{array}{cc} A & B_{-i}\cdot {\hat{u}}_{-i}\left(t\right)\\ 0 & 1 \end{array}]\cdot [\begin{array}{c} x(t)\\ 1 \end{array}]+[\begin{array}{c} B_{i}\\ 0 \end{array}]\cdot [u_{i}\left(t\right)+\eta_{i}\left(t\right)\right] \end{array}$$ (14)

where ${\hat{u}}_{-i}\left(t\right)$ is the contribution of the partner, estimated at the previous trial. This augmented dynamics can be used to calculate the optimal control policy by using the LQG algorithm. The controller has the form of Eq 7 involves a feedback component, which depends on the estimated dyad state. The dummy state variable also results in a feedforward component, which reflects the contribution of the partner. Our implementation of fictitious play only uses the most recent estimate of partner’s input. This is less robust than estimating the distribution of partner inputs over multiple repetitions, but may be adequate for practical purposes.

Wherever possible, we set the model parameters to values that were empirically motivated. When this was not possible/feasible, we used values which resulted (in the H group, taken as reference) in figures of interaction force, minimum via-point distance and leadership indexes which were similar to the experimental observations—see the S1 File for details.

All simulations were performed using MATLAB and Simulink. The simulation results were analyzed in exactly the same way as the experimental results. In particular, for both models we calculated both dyad- and subject-level indicators. The simulation results are summarised in Fig 5.

The model is not meant to exactly reproduce the experimental data, but rather to make general predictions. Different from the score used during experiments, in the model the task is completely specified by a quadratic cost functional which reflects all the instructions that we gave to the participants, including those that were not included in the score—for instance, reaching the target and stopping there. The cost functional also includes an additional essential requirement –minimizing the effort—which is biologically motivated and is implicit in any motor task.

## Supporting information

S1 File Supplementary note with model implementation details. (PDF)

S1 Data Zip file containing data from all indicators and all groups (H,VH, PV)—one file per indicator and per group. Each file has 5 rows (one per dyad) and 156 columns (one per trial).
(ZIP)
