# Supplementary material for: Incomplete information about the partner affects the development of collaborative strategies in joint action
Source: PLoS Comput Biol. 2019 Dec 12;15(12):e1006385. doi: 10.1371/journal.pcbi.1006385 (PMC6907753; doi:10.1371/journal.pcbi.1006385)
Supplement: S1 File — (PDF) [file pcbi.1006385.s001.pdf]

Supplementary Note

Incomplete information about the partner affects the development of collaborative strategies in joint action

Vinil T. Chackochan and Vittorio Sanguineti

Department of Informatics, Bioengineering, Robotics and Systems Engineering
University of Genoa, Italy

This document describes how the general modeling framework, described in the Methods section of the main document is used to run computer simulations of the interaction experiments. The purpose of simulations is to characterise scenarios in which each partner autonomously determines his/her actions, based on a variety of assumptions about his/her knowledge about the dyad, the task and the partner. When using the model we focus on two separate objectives. First, we use the general game-theoretic framework to predict optimal dyad behaviours. Second, we use fictitious play to model how the players develop joint coordination as a result of repeated task performance. We use this model to assess how lack of information about the partner affects the learned strategy.

1 Model summary

We modelled plant behavior and task as a differential non-cooperative game with Gaussian noise and quadratic costs. We also assumed that each player has a state observer which also predicts the partner's actions. Model formulation is described in the main text and is summarized by the following equations:

Plant dynamics: $x(t+1) = A \cdot x(t) + B_1 \cdot [u_1(t) + \eta_1(t)] + B_2 \cdot [u_2(t) + \eta_2(t)]$

and, for each player ($i = 1, 2$):

Sensory system: $y_i(t) = H_i \cdot x(t) + v_i(t)$

Cost function(s): $J_i\left[u_i, u_{-i}\right] = \sum_{t=1}^{T-1} \left[x(t)^T \cdot Q_i(t) \cdot x(t) + u_i(t)^T \cdot R_i(t) \cdot u_i(t)\right] + x(T)^T \cdot Q_i(T) \cdot x(T)$

Optimal controller(s): $u_i(t) = -L_i(t) \cdot x(t)$

State observer(s): $\hat{x}_i^+(t+1) = \hat{x}_i^-(t+1) + K_i(t+1) \cdot [y_i(t+1) - H_i \cdot \hat{x}_i^-(t+1)]$

2 Model implementation

To study how joint coordination is influenced by uncertainty about the goals and actions of their partner, we applied the general computational framework, described in the main paper, to a sensorimotor version of classic battle of sexes game. Partners were mechanically connected through a compliant virtual spring and they have partly conflicting goals – reaching the same target through different via-points.

2.1 Dyad dynamics

In our simulated dyad movements, we approximated each player's upper limb and robot dynamics as a point mass m_i, $i = 1, 2$:

$$m_i \ddot{p}_i = f_i + k \cdot (p_{-i} - p_i) - b \cdot \dot{p}_i + m_i g \tag{1}$$

where $p_i(t)$ and $p_{-i}(t)$ are the hand position vectors of, respectively, player i and his/her partner $-i$; m_i is the player's mass, $f_i(t)$ is the muscle-generated force vector. We also assumed that each player is subjected to gravity and to a small viscous force accounting for the damping caused by muscles and soft tissue. In all simulations, consistent with the actual experiments – see the main paper – we took $m_1 = m_2 = 2$ kg, $b = 10$ N s/m and $k = 150$ N/m.

As in [1], we modelled the dynamics of muscle force generation as a second order system:

$$\tau^2 \ddot{f}_i + 2\tau \dot{f}_i + f_i = u_i \tag{2}$$

where $u_i(t)$ is the activation vector, which is taken as system's input, and τ is the activation time constant, which we set to $\tau = 40$ ms. By defining the overall state vector as $x = [p_1^T, \dot{p}_1^T, f_1^T, \dot{f}_1^T p_2^T, \dot{p}_2^T, f_2^T, \dot{f}_2^T]^T$, the dyad dynamics can be rewritten in state-space form:

$$\dot{x} = A_c \cdot x + B_{c1} \cdot (u_1 + \eta_1) + B_{c2} \cdot (u_2 + \eta_2) + c_c \tag{3}$$

where

$$A_c = \begin{bmatrix} 0_2 & I_2 & 0_2 & 0_2 & 0_2 & 0_2 & 0_2 & 0_2 \\ -k/m_1 & -b/m_1 & 1/m_1 & 0_2 & k/m_1 & 0_2 & 0_2 & 0_2 \\ 0_2 & 0_2 & 0_2 & I_2 & 0_2 & 0_2 & 0_2 & 0_2 \\ 0_2 & 0_2 & -I_2/\tau^2 & -2I_2/\tau & 0_2 & 0_2 & 0_2 & 0_2 \\ 0_2 & 0_2 & 0_2 & 0_2 & 0_2 & I_2 & 0_2 & 0_2 \\ k/m_2 & 0_2 & 0_2 & 0_2 & -k/m_2 & -b/m_2 & 1/m_2 & 0_2 \\ 0_2 & 0_2 & 0_2 & 0_2 & 0_2 & 0_2 & 0_2 & I_2 \\ 0_2 & 0_2 & 0_2 & 0_2 & 0_2 & 0_2 & -I_2/\tau^2 & -2I_2/\tau \end{bmatrix}$$

$$[B_{c1}|B_{c2}] = \begin{bmatrix} 0_2 & 0_2 \\ 0_2 & 0_2 \\ 0_2 & 0_2 \\ I_2/\tau^2 & 0_2 \\ 0_2 & 0_2 \\ 0_2 & 0_2 \\ 0_2 & 0_2 \\ 0_2 & I_2/\tau^2 \end{bmatrix}$$

$$c_c = \begin{bmatrix} 0_2 \\ 0 \\ -g \\ 0_2 \\ 0_2 \\ 0_2 \\ 0 \\ -g \\ 0_2 \\ 0_2 \end{bmatrix}$$

where we defined $I_2 = \begin{bmatrix} 1 & 0 \\ 0 & 1 \end{bmatrix}$ and $0_2 = \begin{bmatrix} 0 & 0 \\ 0 & 0 \end{bmatrix}$.

If η_1 and η_2 are process noise sources (one per player), assumed to be Gaussian with covariance Σ_i^η, Eq. 3 can be rewritten as:

$$\dot{x} = A_c \cdot x + B_{c1} \cdot u_1 + B_{c2} \cdot u_2 + c_c + w_1 + w_2 \tag{4}$$

where $w_i = B_i \eta_i$, with variance $\Sigma_i^w = B_i \Sigma_i^\eta B_i^T$.

From Eq. 5 it is possible to calculate the state vector x^{eq} and the control inputs u_1^{eq} and u_2^{eq} which balance gravity forces:

$$A_c \cdot x^{eq} + B_{c1} \cdot u_1^{eq} + B_{c2} \cdot u_2^{eq} = -c_c \tag{5}$$

We then set $x = x - x^{eq}$, $u_1 = u_1 - u_1^{eq}$ and $u_2 = u_2 - u_2^{eq}$, so that the term c_c disappears from the model.

For simulation purposes, the model equations were discretised by using a first-order hold method, with a sampling rate $\Delta t = 1$ ms over a movement duration of $T = 2$ s. We then obtained:

$$x(t+1) = A \cdot x + B_1 \cdot u_1 + B_2 \cdot u_2 + w_1 + w_2 \tag{6}$$

After model discretisation, we added three extra state variables to store information about the position of the target x_T and of the two via-points, x_{VP_1} and x_{VP_2} so that the new state is: $X = [x, x_T, x_{VP_1}, x_{VP_2}]^T$ – a 22-dimensional vector.

In all simulations we took $\Sigma_i^u = \mathrm{diag}(1,1)\,\mathrm{N}^2$, identical for both players.

2.2 Task and cost functionals

The task (reaching a target through a via-point) was specified in terms of the following cost functionals ($i = 1, 2$):

$$\begin{aligned} J_i[u_1, u_2] = & w_p \cdot \|x_T - x_i(T)\|^2 + \\ & w_v \cdot \|\dot{x}_i(T)\|^2 + \\ & w_{vp} \cdot \|x_{VP_i} - x_i(tc_i)\|^2 + \\ & w_f \cdot \frac{1}{T} \sum_{t=1}^{T} \|x_{-i}(t) - x_i(t)\|^2 + \\ & r \cdot w_u \cdot \frac{1}{T} \sum_{t=1}^{T} u_i(t)^2 \end{aligned} \tag{7}$$

The cost functional has five terms. The first two terms enforce stopping on target at the end of the movement (small endpoint error, small endpoint velocity). The third term reflects the requirement to pass through the via-point (small via-point distance). The fourth term accounts for keeping the interaction force (proportional to the distance between players) low throughout the movement. The last term penalises the effort incurred during the movement.

The weight coefficients determine the relative importance of the corresponding constraint. We set these weights by assuming (Bryson's rule) a maximum acceptable displacement (in the via-point and in the final target) equal to, respectively, the radius of the via-point ($x_{VP}^{max} = 2.5$ mm) and that of the target ($x_T^{max} = 5$ mm). We then set $w_{vp} = 1/(x_{VP}^{max})^2$ and $w_p = 1/(x_T^{max})^2$. We made similar normalisations for the 'velocity' weight, w_v – calculated by assuming a maximum acceptable speed at the target of 5 mm/s – for the maximum inter-player distance (25 mm) and the maximum activation (15 N).

The scalar coefficient r – the only free parameter in the model – specifies the trade-off between task-related accuracy and effort. With $r \gg 1$, the optimal strategy is not moving at all. With $r \ll 1$, the optimal strategy pays little attention to effort requirements. In all simulations we used r=1.

The cost functional reflects all the instructions that we gave to the participants, including those that were not included in the score displayed to participants during the experiments – for instance, reaching the target and stopping there. The cost functional also includes an additional essential requirement – minimizing the effort – which is biologically motivated and is implicit in any motor task. Both the score displayed during the experiment and the cost functional include a term related to via-point distance and another related to average inter-player distance, but with some differences. First, the cost functional is a quadratic form and the score function is a sigmoid. In the cost functional these terms are expressed as square errors, whereas in the score they are expressed as absolute errors (also the sigmoid shape) The relative weights of these terms are also different. In the score we set the ratio interaction error/via-point error= 0.5, whereas in the cost functional the ratio is much lower (interaction error/via-point error = 0.01). However, the effort minimization term also indirectly contributes to reducing the inter-player distance. Therefore, the cost functional used in simulation can be considered as functionally equivalent to the score function used in the experiments.

2.2.1 Calculation of optimal via-point crossing times

In the cost functional of Eq. 7, the times of crossing of the via-points, tc_1 and tc_2 are themselves part of the optimization. To calculate the optimal crossing times, we systematically varied tc_1 and tc_2 (between 10% and 90% of total duration – set to 2 s in all simulations) over a square grid. For each crossing time pair, we calculated the average magnitude of the optimal cost for both players in the dyad, J_1 and J_2. We then smoothed both the J_1 and J_2 mappings using a radial basis functions approximation. In all subsequent simulations, as the optimal via-point crossing times tc_i we then took the values that corresponded to the Nash equilibrium (intersection of the reaction lines) calculated in the smoothed pair of cost functionals; see Figure A.

The Figure clearly indicates that there are indeed two cost function minima, corresponding to crossing VP_1 first and then VP_2 ($tc_1 < tc_2$) and vice versa. Given that VP_1 is closer to the start position than VP_2, the first solution requires less effort and is therefore the global optimum.

We ended up with crossing time values of, respectively, 36% and 71% of the total movement duration (Nash condition) and 28% and 64% (No-partner condition). Hence the optimal crossing time values are slightly different in both via-points in the two extreme conditions (Nash and No-partner). To simplify calculations, in the subsequent fictitious playing simulations we used constant crossing time values (those corresponding to the Nash condition), which is indeed sub-optimal as they are expected to change at each iteration. To test the impact of this simplifying assumption, we ran additional simulations using the optimal crossing times corresponding to the no-partner conditions. We found slightly different values of the final minimum distance, interaction forces and leadership indices, but the main prediction (when information increases the learned strategy comes closer to Nash equilibrium) did not change.

2.3 Feedback controllers

2.3.1 Nash controllers

The optimal Nash feedback controllers can be determined through the following iterative algorithm [2]:

$Z_i(T) \leftarrow Q_i(T)$

for $t \leftarrow (T-1), 0$ **do**

solve for $i = 1, 2$**:**

$$\left[R_i(t) + B_i^T \cdot Z_i(t+1) \cdot B_i\right] \cdot L_i(t) + \left[B_i^T \cdot Z_i(t+1) \cdot B_{-i}\right] \cdot L_{-i}(t) = B_i^T \cdot Z_i(t+1) \cdot A$$

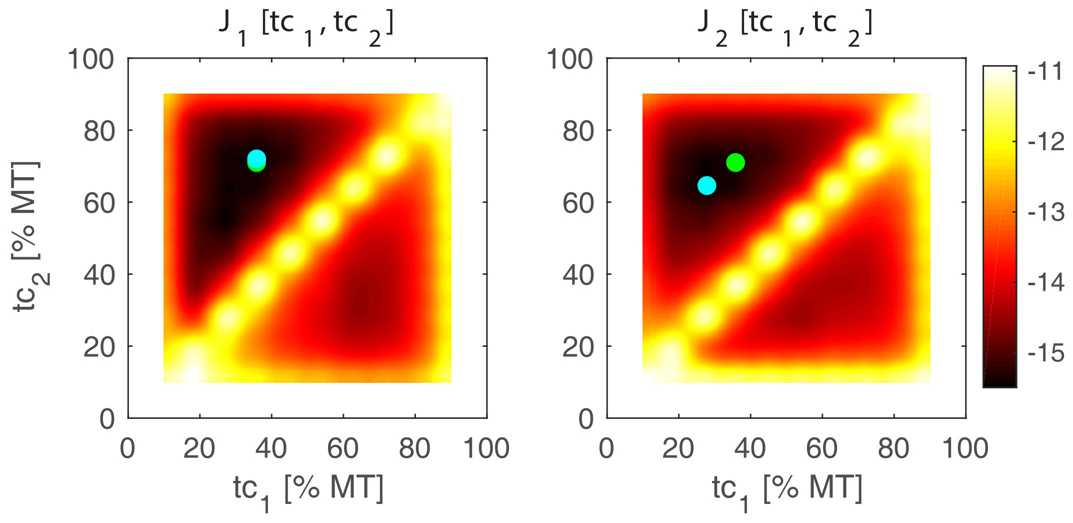

Figure A: Crossing time pairs calculated for the partners' smoothed cost functions J_1, J_2. Optimal pairs for each cost function (cyan) and Nash equilibria (green)

$F(t) \leftarrow -B_1 \cdot L_1(t) - B_2 \cdot L_2(t)$
$Z_i(t) \leftarrow Q_i(t) + F(t)^T \cdot Z_i(t+1) \cdot F(t) + L_i(t)^T \cdot R_i(t) \cdot L_i(t)$
end for

In the above equation and in all the following, i denotes a player and $-i$ denotes his/her partner.

2.3.2 Optimal 'no-partner' controllers

A second (sub-optimal) scenario is represented a the situation in which each player assumes that his/her partner is inactive, i.e. $u_{-i}(t) = 0$.

In this case, the optimal controllers are calculated independently, as two separate LQG optimal control problems:

$Z_i(T) \leftarrow Q_i(T)$
for $t \leftarrow (T-1), 0$ **do**
$L_i(t) = \left[R_i(t) + B_i^T \cdot Z_i(t+1) \cdot B_i\right]^{-1} B_i^T \cdot Z_i(t+1) \cdot A$
$Z_i(t) \leftarrow Q_i(t) + L_i(t)^T \cdot R_i(t) \cdot L_i(t) + [A - B_i \cdot L_i(t)]^T \cdot Z_i(t+1) \cdot [A - B_i \cdot L_i(t)]$
end for

The above algorithm is separately applied to both players, i.e. for $i = 1, 2$.

2.4 Sensory systems

Each player has his/her own sensory system $y_i(t)$, which provides information about the dyad state. Reliability of the sensory information is determined by the magnitude of the sensory noise, $v_i(t)$, assumed to be Gaussian. The sensory system of each player is described by:

$$y_i(t) = H_i \cdot x(t) + v_i(t) \quad (8)$$

The structure of the H_i matrix depends on the available sensory information. In the H and VH groups (see the main paper) the sensory information is defined as $y_i = [p_i, \dot{p}_i, k(p_{-i} - p_i), x_T, x_{VP_i}]^T$. For Player 1

we have:

$$H_1 = \begin{bmatrix} I_2 & 0_2 & 0_2 & 0_2 & 0_2 & 0_2 & 0_2 & 0_2 & 0_2 & 0_2 & 0_2 \\ 0_2 & I_2 & 0_2 & 0_2 & 0_2 & 0_2 & 0_2 & 0_2 & 0_2 & 0_2 & 0_2 \\ -k \cdot I_2 & 0_2 & 0_2 & 0_2 & k \cdot I_2 & 0_2 & 0_2 & 0_2 & 0_2 & 0_2 & 0_2 \\ 0_2 & 0_2 & 0_2 & 0_2 & 0_2 & 0_2 & 0_2 & 0_2 & I_2 & 0_2 & 0_2 \\ 0_2 & 0_2 & 0_2 & 0_2 & 0_2 & 0_2 & 0_2 & 0_2 & 0_2 & I_2 & 0_2 \end{bmatrix} \tag{9}$$

A similar expression is found for H_2.

The participants in the PV group are assumed to also see their partner's position so that the sensory information is defined as $y_i = [p_i, \dot{p}_i, p_{-i}, \dot{p}_{-i}, k(p_{-i}-p_i), x_T, x_{VP_i}]^T$ and H_1 and H_2 are modified accordingly.

The measurement noise is assumed to be Gaussian with variance:

$$\Sigma_i^v = \mathrm{diag}(\sigma_x^2, \sigma_x^2, \sigma_{xd}^2, \sigma_{xd}^2, \sigma_f^2, \sigma_f^2, \sigma_x^2, \sigma_x^2, \sigma_x^2, \sigma_x^2) \tag{10}$$

We set $\sigma_x^2 = 1.7^2\,\mathrm{mm}^2$, $\sigma_{xd}^2 = 35^2\,\mathrm{mm}^2/\mathrm{s}^2$. For the H and VH group, we respectively set $\sigma_f^2 = 2^2\,\mathrm{N}^2$ and $\sigma_f^2 = 0.05^2\,\mathrm{N}^2$.

2.5 Partner model

Partner's control input was estimated as part of the state observer. We made the prior assumption that partner input is described by a low-pass filtered white noise:

$$u_{-i}(t+1) = A_u \cdot u_{-i}(t) + \varepsilon_{-i}(t) \tag{11}$$

In all simulations, we set $A_u = 1$ and $\Sigma_{-i}^{\varepsilon} = 1\ \mathrm{N}^2$.

References

1. Todorov E, Jordan MI. Optimal feedback control as a theory of motor coordination. Nature Neuroscience. 2002;5(11):1226–35. doi:10.1038/nn963.

2. Başar T, Olsder GJ. Dynamic noncooperative game theory. vol. 23. 2nd ed. SIAM; 1999.
